# The therapy frequency of antibiotics and phenotypical resistance of *Escherichia coli* in calf rearing sites in Germany

**DOI:** 10.3389/fvets.2023.1152246

**Published:** 2023-05-18

**Authors:** Roswitha Merle, Susann Weise, Lisa Gorisek, Jorinde Baer, Caroline Robé, Anika Friese, Katharina Charlotte Jensen

**Affiliations:** ^1^Department Veterinary Medicine, Institute for Veterinary Epidemiology and Biostatistics, Freie Universität Berlin, Berlin, Germany; ^2^Department Veterinary Medicine, Institute of Animal and Environmental Hygiene, Freie Universität Berlin, Berlin, Germany

**Keywords:** antimicrobials, calves, AMR score, therapy frequency, antibiotics use

## Abstract

**Introduction:**

The association between antibiotic use and the occurrence of resistant bacteria is a global health problem and is subject to enormous efforts at national and international levels. Within the scope of the study “KAbMon”, the resistance situation as well as the use of antibiotics in calf rearing farms in Germany was investigated. We hypothesized that the levels of resistance are associated with certain calf keeping farm types, such as pre-weaned calf farms, animal groups, and therapy frequency.

**Methods:**

In total, 95 calf keeping farms were visited between October 2019 and April 2021. At each farm, up to three pooled fecal samples (10 freshly released feces each) were collected. One sample was taken in the youngest calf group, another in the oldest calf group, and one in the hospital box, if available. *Escherichia coli* was isolated from non-selective MacConkey agar. The therapy frequency reflects the average number of treatment days per calf in a half-year, while the resistance score is the sum of the relative minimum inhibitory concentration per substance over all 10 tested substances.

**Results:**

The 1781 isolates from 178 samples showed high resistance rates against sulfamethoxazole (82%), tetracycline (49%), and ampicillin (40%). High resistance scores were mainly found in pre-weaned calf farms (purchasing calves from 2  weeks of life) and in the youngest animals. The therapy frequency showed an almost linear relationship with the resistance scores, and the age at purchase was negatively related to the resistance score.

**Discussion:**

The high use of antimicrobials of young calves might be associated with a high risk for infectious diseases and might indicate that the current system of crowding 14-day-old calves from different farms in one group is not optimal. Further efforts are necessary to educate and motivate the calf keepers to ensure highest levels of hygiene and management as well as animal welfare conditions and to increase animal health.

## 1. Introduction

The association between antibiotic use and the occurrence of resistant bacteria is a global health problem and is subject to enormous efforts at the national and international levels (1–3). Farm animal husbandry and the veterinary sector are regarded as some of the main drivers, and thus, are supposed to reduce its antibiotic use (4, 5). In Germany, national antibiotic monitoring, including the concept of minimizing antibiotic use in farm animals, has been in force since 2014 (6). Animal keepers for fattening purposes have to report their antibiotic use to a national database twice per year (6). Therapy frequency (TF) is calculated for each farm, and farms above certain thresholds (median and 3rd quartile) need to take action to increase animal health and reduce TF (6).

**Figure 1:**
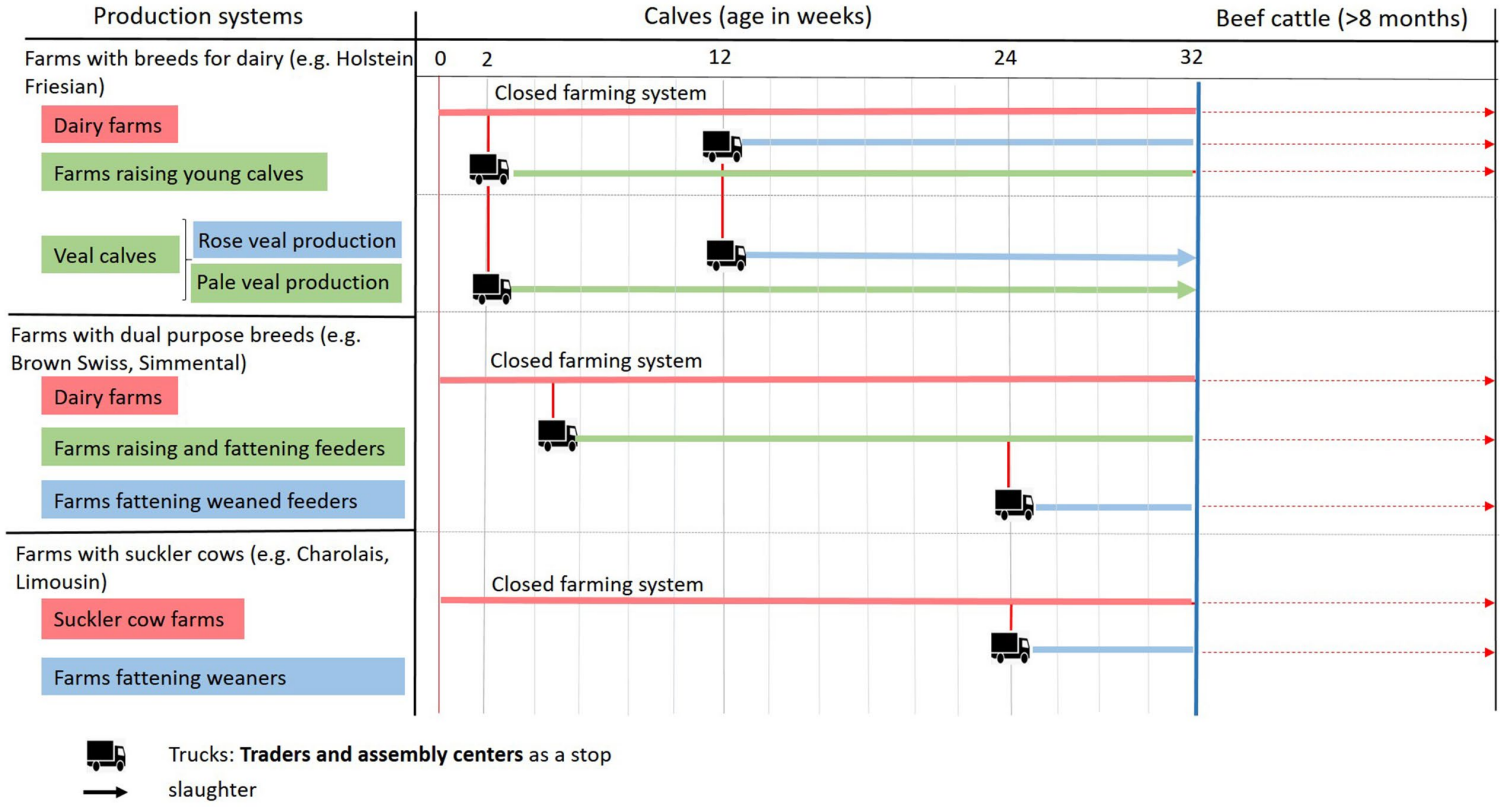
Structure of calf raising systems in Germany. Red, dairy or suckler cow farms (closed farming systems); green, farms raising pre-weaned calves; blue, farms raising weaned calves.

All fattening calves are compiled within the group “fattening calves up to 8 months of age,” although there are huge differences in the need for antibiotic treatment between different farm types, e.g., dairy farms and calf rearing farms (6). Since the beginning of the monitoring program, the median TF for calves was 0, meaning that at least half of the farms reported no antibiotic use in their calves. However, the third quartile fluctuates around 2, in contrast to fattening bulls older than 8 months, where the third quartile is 0 (7). In Germany, there are about 53,000 dairy farms in which all newborn calves are raised at least up to 14 days of life (8). After 14 days, calves were raised as future dairy cows or sold to specialized calf-rearing farms (Figure 1). Pre-weaned calf farms assemble 14-day-old calves from dairy farms and raise them until slaughter (9, 10). Approximately 300 farms are members of the German veal meat group (11). In addition, there are farms specialized for fattening calves that are slaughtered as cattle at the age of 18 months. It is well known that pre-weaned calves after transport to the rearing farms are of high risk for infectious diseases and therefore, often need antibiotic treatment (12). Thus, the health risk situation between calves in dairy farms and those in rearing farms is quite different, and the antibiotic use between these types of calf husbandry cannot be compared fairly. Therefore, in the future, the group “fattening calves up to 8 months of age” should be split into several groups to ensure comparability (13).

The resistance situation in calf-keeping farms and in contaminated foods derived from calves is alarming. In a report on zoonoses monitoring by the Federal Office of Consumer Protection and Food Safety (BVL), a prevalence of extended-spectrum beta-lactamase and plasmid-mediated AmpC beta-lactamase (ESBL-/pAmpC-) producing *Escherichia coli* (*E. coli*) of 70.8% was found in 407 cecum samples from calves in slaughterhouses, and 3.4% of investigated fresh meat samples from retail were contaminated with ESBL-/AmpC-producing *E. coli* (14).

*E. coli* is a ubiquitous bacterium some strains of which are pathogenic, others commensal. They are used as indicator bacteria for the assessment of the resistance situation, because they are part of the healthy gut flora and are distributed easily to other animals, animal species and even humans.

The research project “KAbMon” (monitoring of antibiotics use in calves in different rearing types in Germany) monitored antimicrobial usage in different groups of calf keeping farms. The German Federal Ministry for Nutrition and Agriculture funded this study (support code 2818HS014). Within the scope of the study “KAbMon,” among other things, the resistance situation as well as the use of antibiotics in calf rearing farms in Germany was investigated. We hypothesized that the levels of resistance are associated with certain farm types, such as pre-weaned calf farms, animal groups, and TF.

## 2. Materials and methods

The study farms were stratified for three different farm types (Table 1) as well as for the regions of North Germany (Lower Saxony, North Rhine-Westphalia, and Schleswig-Holstein), East Germany (Brandenburg, Mecklenburg–West Pomerania, and Thuringia), and South Germany (Bavaria). The sample size calculation was based on the hypothesis that there were significant differences between farm types in the percentage of isolates with multiple resistance. Assuming a difference between the three farm types of 20% (50% vs. 70%), a power of 80%, and alpha of 5% requires a group size of 19 farms per group (15). To account for further risk factors, such as region and breed, and multiple comparisons between farm types, we added 4 × 15% = 60%, i.e., 31 farms per farm type (16). Based on the different number of farms per farm type and region, the distribution of the farms was as presented in Table 1.

**Table 1:** Definition of the farm types and number of farms and samples per farm type.

Farm type	Definition	Sample size
Dairy farms (closed farming systems)	Dairy or suckler cow herds	44 samples from 26 farms
Pre-weaned calf farms	Pre-weaned calf raiser/veal calves purchased <10-week old calves	90 samples from 43 farms
Weaned calf farms	Fattening (weaned) calves purchased ≥10-week-old calves	44 samples from 26 farms

Farms were recruited by direct contact of their veterinarian for pre-weaned and weaned calf farms. Dairy farms were recruited by announcements at farmers’ assemblies. In total, 95 calf keeping farms were visited between October 2019 and April 2021 (Table 1). At each farm, up to three pooled fecal samples (each pooled sample was composed of 10 freshly released feces) were collected. One of the pooled samples was taken from the youngest calf and the other from the oldest calf group. If available, a third pooled sample was collected from the hospital boxes. The actual age of the calves was unknown and is described in the results part.

### 2.1. Therapy frequency

To record the use of antimicrobial substances, drug application and dispensing records since January 1, 2018 were either transmitted electronically from the associated veterinary practice (written consent by farmers) or scanned and digitized manually. The treatment frequencies were calculated for half-years from January 2018 to February 2020 according to the common standard (6) (Table 2). For the analysis, the therapy frequency of the farm visit’s half-year was used, i.e., “current therapy frequency.”

**Table 2:** Formulas for the calculation of the treatment frequency.

Definition	Formula
Sum of animal days	*Animal days* $=Days_{Animal1}$ + $\;Days_{Animal2}$ + $Days_{Animal3}+$ ... + $Days_{Animaln}$
Average number of animals kept	*Average number* $=\frac{Animaldays}{Daysof\;a\;halfyear}$
Treatment frequency	$Treatmentfrequency=\frac{Numberoftreatedanimals.Daysundertreatment.Numberofactiveingredients}{Averagenumberofanimalskept}$

### 2.2. Laboratory analyzes

After microbiological processing of the pooled fecal samples on non-selective MacConkey agar No. 3 (Fisher Scientific GmbH, Schwerte, Germany), 10 randomly selected isolates of *E. coli* per sample were confirmed using matrix-assisted laser desorption time of flight (MALDI-TOF Microflex® LT and Biotyper database®; Bruker Daltonics, Bremen, Germany), and antimicrobial susceptibility was tested with minimum inhibitory concentration (MIC) using the EUVSEC format according to BVL zoonoses monitoring with controls according to EUCAST. MIC values were categorized using epidemiological cutoff values following 2013/652/EU. If no cutoff values were available, the respective EFSA recommendations were applied.

The following substances were tested: ampicillin (AMP), azithromycin (AZM), ceftazidime (CAZ), ciprofloxacin (CIP), chloramphenicol (CMP), colistin (COL), cefotaxime (CTX), gentamicin (GEN), meropenem (MER), nalidixic acid (NAL), sulfamethoxazole (SMO), tetracycline (TET), tigecycline (TGC), and trimethoprim (TRP).

In addition, the minimum inhibitory concentration (MIC) that inhibited growth of 50% (MIC50) or of 90% (MIC90) of the isolates were calculated.

### 2.3. Resistance score

The antimicrobial resistance (AMR) score was calculated as follows (17). For each substance, the number of tested concentrations including the categories “below lowest tested concentration” and “above highest tested concentration” was ranked. For example, ampicillin was tested at seven different concentrations: (<1) – 1 – 2 – 4 – 8 – 16 – 32 – 64 – (>64) μg/mL. This resulted in nine possible results, with <1 = range 0 and >64 = range 8. Isolates <1 μg/mL were given 0/8 = 0, and isolates >64 μg/mL were given 8/8 = 1. These values were summed for all substances per isolate.

Meropenem and tigecycline were not considered because all isolates were below the lowest tested concentrations. Ceftazidim was also not considered because of its close relation to cefotaxime; nalidixic acid was excluded for the same reason (related to ciprofloxacin). Thus, only 10 substances were included; the minimum AMR score was 0, and the maximum was 10.

### 2.4. Data analysis

Data were analyzed using R version 4.2.1 (18) and RStudio version 2022.07.1 (19). Descriptive analyzes were carried out using the R packages tidyverse (20), readxl (21), and janitor (22). Generalized additive mixed models were fitted using lme4 (23) and gamm4 (24), allowing polynomial fitting of the relationship between the current therapy frequency and AMR score, as well as between the median age at purchase and AMR score. For this analysis, the AMR score was used at the sample level, that is, the mean AMR score for all isolates per sample.

## 3. Results

A total of 178 fecal samples were collected from 95 farms (Table 1). Thereof, 82 samples were derived from the youngest animals, 78 from the oldest animals, and only 18 from the hospital box (17 farms, one dairy farm, 12 pre-weaned calf farms, and 4 weaned calf farms). Sixty-seven farms were located in the Northern region (118 samples), 11 in the Eastern region (24 samples), and 17 in the Southern region (36 samples). All farms that raised weaned calves were located in the Northern region, and pre-weaned calf farms were underrepresented in the Eastern region (Table 3), but the distribution of animal groups between the farm types and between the regions was almost even.

**Table 3:** Number of samples investigated per farm type and region.

Region	Dairy farms	Pre-weaned calf farms	Weaned calf farms	Total
North	19	55	44	118
East	14	10	0	24
South	11	25	0	36
Total	44	90	44	178

In pre-weaned calf farms, the median age of purchase of the youngest animals was 36 days (interquartile range: 20–41.5), and the oldest animals were on average 208 days old (median, 145–528). In weaned calf farms, the animals were purchased at a median of 149 days (115–174 days) and disposed at a median age of 548.5 days (255–617 days). Only animals up to 243 days of life were considered in the study because older animals do not fall into the category “calves up to 8 months.” Since in dairy farms, the calves were born in that farm and often were not removed from the farm before the age of 8 months, neither age at purchase nor at disposal can be described.

### 3.1. Minimum inhibitory concentration per substance

In total, 1781 isolates were investigated for antimicrobial susceptibility (farm types: 440 from dairy farms, 901 from pre-weaned calves, 440 from weaned calf farms; age groups: 820 from the youngest age group, 781 from the oldest age group, and 180 from the hospital box). The distribution of MIC values for each substance and isolate is shown in Table 4. Regarding SMO, more than 50% of the isolates had MIC >1,024, i.e., the highest tested concentration. MIC 50 values below the lowest tested concentrations were found for CAZ, CMP, COL, CTX, GEN, MER, NAL, and TGC.

**Table 4:** Antimicrobial susceptibility of *E. coli* isolates derived from non-selective agar.

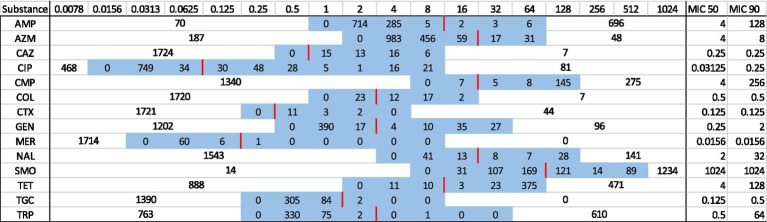

Number of isolates per MIC value (μg/ml). Blue filled boxes represent the tested values, unfilled boxes are values below lowest tested MIC concentration or above highest tested MIC concentration. Red lines represent the cut-off values following the 2013/652/EU or EFSA recommendation (AZM). MIC 50 and MIC 90 values are given in the two right columns. AMP, Ampicillin; AZM, azithromycin; CAZ, ceftazidim; CIP, ciprofloxacin; CMP, chloramphenicol; COL, colistin; CTX, cefotaxim; GEN, gentamicin; MER, meropenem; NAL, nalidixic acid; SMO, sulfamethoxazol; TET, tetracyclin; TGC, tigecyclin; TRP, trimethoprim. (*n* = 1781).

### 3.2. Frequency of resistant isolates per antimicrobial substance

The results of the qualitative assessment of resistance data are presented in Table 5. SMO showed the highest percentage of resistant isolates (82%), followed by TET (49%), AMP (40%), and TRP (34%). 68 (3.8%) of the isolates were resistant to at least one of the cephalosporins, CTX, and CAZ. Resistance rate against COL was 2.1%, whereas more isolates showed resistance to CIP (16%).

**Table 5:** Absolute numbers and relative frequency of resistant isolates per antimicrobial substance.

Substance	Number	Percentage
AMP	707	39.7
AZM	96	5.4
CAZ	57	3.2
CIP	230	15.5
CMP	433	24.3
COL	38	2.1
CTX	60	3.4
GEN	172	9.7
MER	0	0.0
NAL	184	10.3
SMO	1,458	82.0
TET	572	49.0
TGC	0	0.0
TRP	611	34.3

AMP, Ampicillin; AZM, azithromycin; CAZ, ceftazidim; CIP, ciprofloxacin; CMP, chloramphenicol; COL, colistin; CTX, cefotaxim; GEN, gentamicin; MER, meropenem; NAL, nalidixic acid; SMO, sulfamethoxazol; TET, tetracyclin; TGC, tigecyclin; TRP, trimethoprim. (*n* = 1781).

For the following analyzes, the results of the substances CAZ, MER, TGC, and NAL were excluded because they were either all negative (MER, TGC), or were correlated to other tested substances (CAZ to CTX, NAL to CIP). Moreover, 249 (14%) of the isolates were susceptible, 603 (34%) were resistant to one substance, 144 (8%) against two substances, and the remaining 44% of isolates showed resistance to three or more substances, thereof 90 to eight or nine (extensive drug resistant), but none to all 10 substances (pan drug resistant). SMO, TET, and AMP occurred in most of the combinations. Fifty-three isolates (3.0%) were phenotypically ESBLs (resistant to AMP, CAZ, and CTX).

### 3.3. Antimicrobial resistance score

#### 3.3.1. Antimicrobial resistance score per farm type

**Figure 2:**
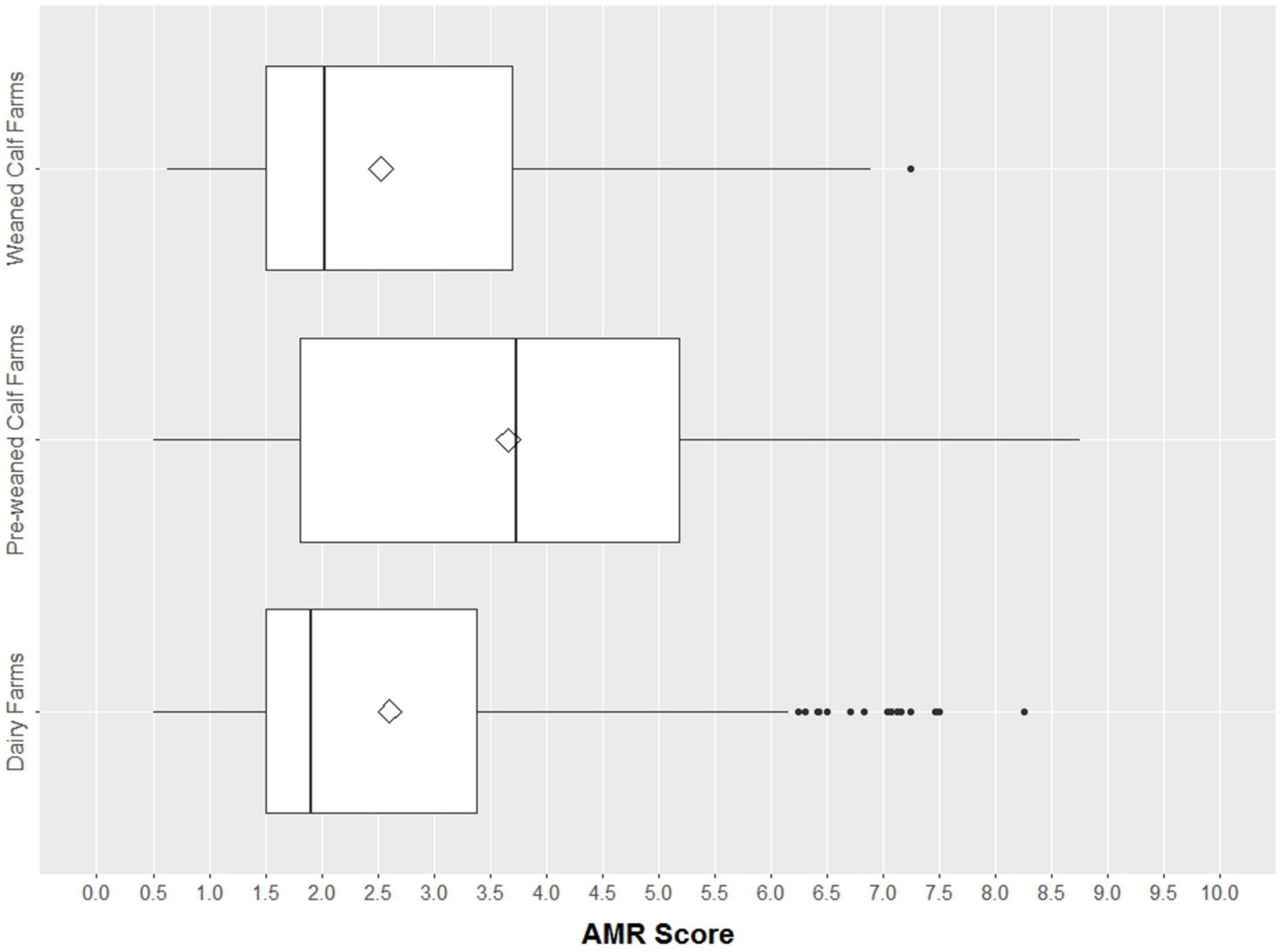
Boxplots of AMR score per isolate in different farm types (*n* = 1781). The box represents the interquartile range (central 50% of the data), the solid vertical line represents the median, and ◊ represents the mean. Dots are >1.5 standard deviations from the mean.

The AMR scores ranged overall from 0.25 to 8.75. Thus, no sample reached a minimum of 0 or maximum of 10.0. The distribution varied between farm types, with higher values in pre-weaned calf farms (Figure 2).

#### 3.3.2. Antimicrobial resistance score per animal group

**Figure 3:**
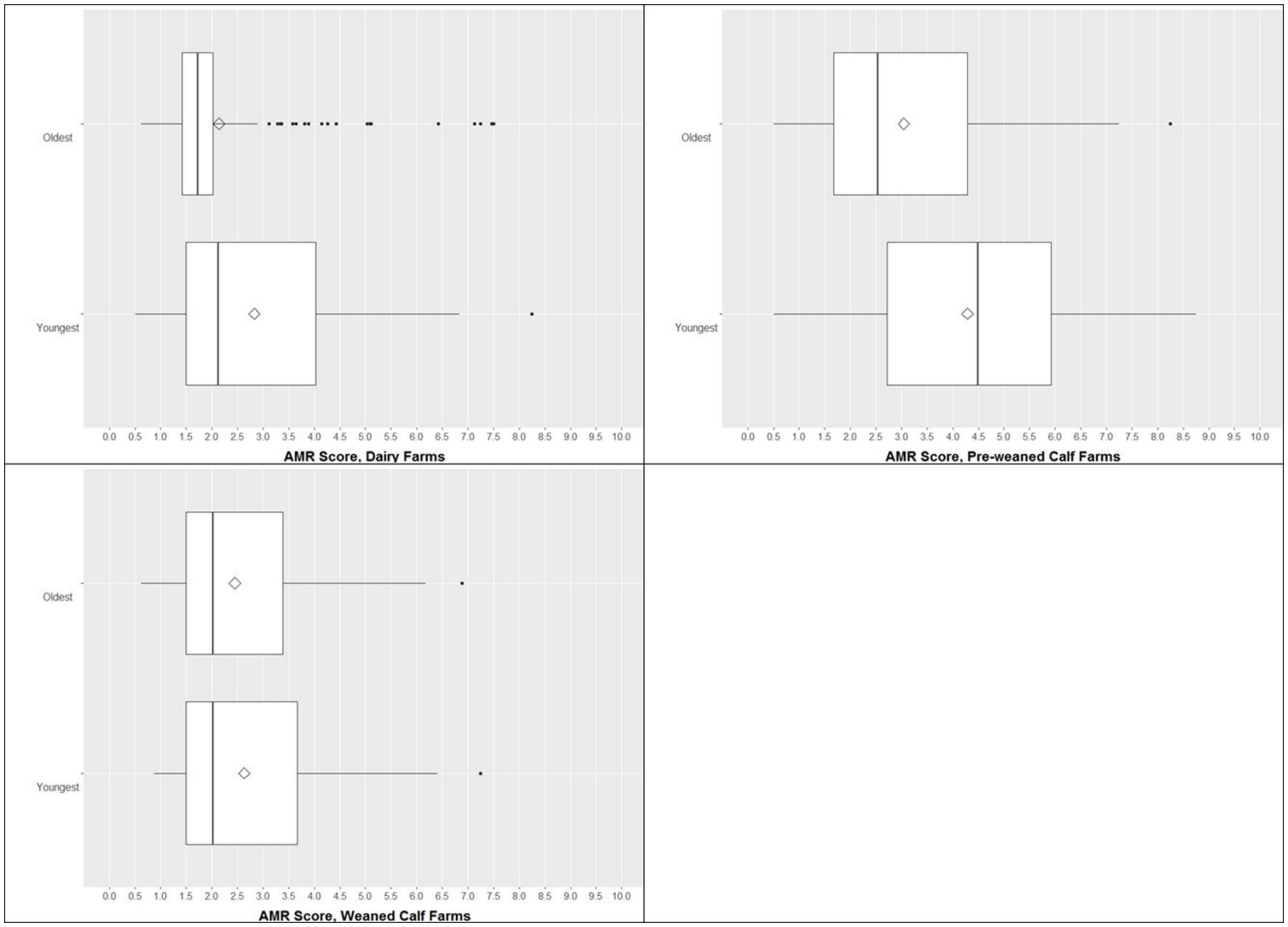
Boxplots of AMR score in the youngest and the oldest animal groups of dairy farms [*n* = 430 isolates (230 youngest, 200 oldest)], pre-weaned calf farms [*n* = 781 isolates (400 youngest, 381 oldest)], and weaned calf farms [*n* = 390 isolates (190 youngest, 200 oldest)]. The box represents the interquartile range (central 50% of the data), the solid vertical line represents the median, and ◊ represents the mean. Dots are >1.5 standard deviations from the mean.

In dairy and pre-weaned calf farms, the average AMR score was higher in the youngest animals (median 3.9) than in the oldest ones (median 2.0; Figure 3). This effect could not be observed in the weaned calf farms, since the median AMR values in each group were approximately 2.0. However, on weaned farms, the youngest group was older than the youngest group on dairy and pre-weaned calf farms. The range of AMR scores in the youngest pre-weaned calves was very broad, indicating large differences between the farms. Although the median was much smaller in the oldest animals, the range remained wide. On dairy farms, the oldest animals had a median AMR score below 2, and the range was rather small, although outliers ranged up to 7.5 (Figure 3).

#### 3.3.3. Antimicrobial resistance score in hospital boxes

**Figure 4:**
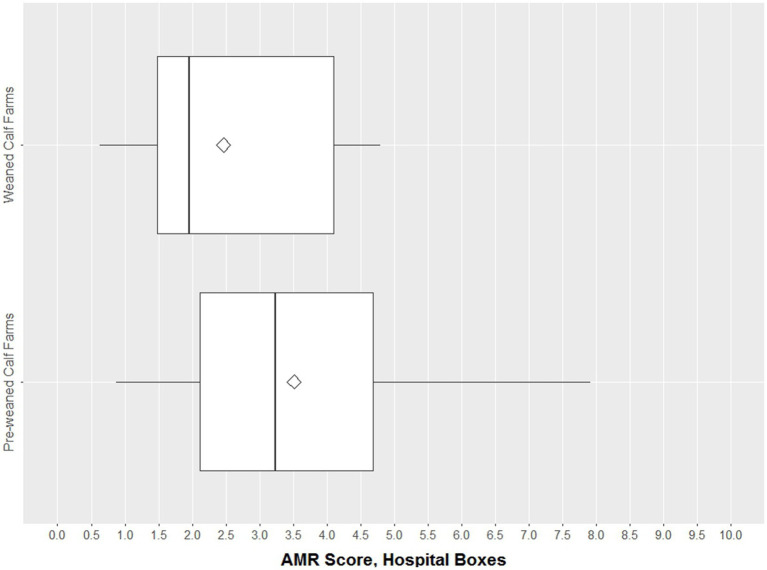
Boxplots of AMR score in the hospital boxes [*n* = 170 isolates (120 pre-weaned calf, 50 weaned calf farms)]. The box represents the interquartile range (central 50% of the data), the solid vertical line represents the median, and ◊ represents the mean. Dots are values >1.5 standard deviations from the mean.

Neither in weaned calf farms (median 2.0 in all groups) nor in the pre-weaned calf farms (median 3.2 compared to 4.5 and 2.5 in the animal group), a trend toward higher AMR scores in the hospital boxes could be observed (Figure 4). Since the 10 isolates from hospital boxes in the dairy farms originated from the same farm, this result is not displayed here.

#### 3.3.4. Antimicrobial resistance score and TF

**Figure 5:**
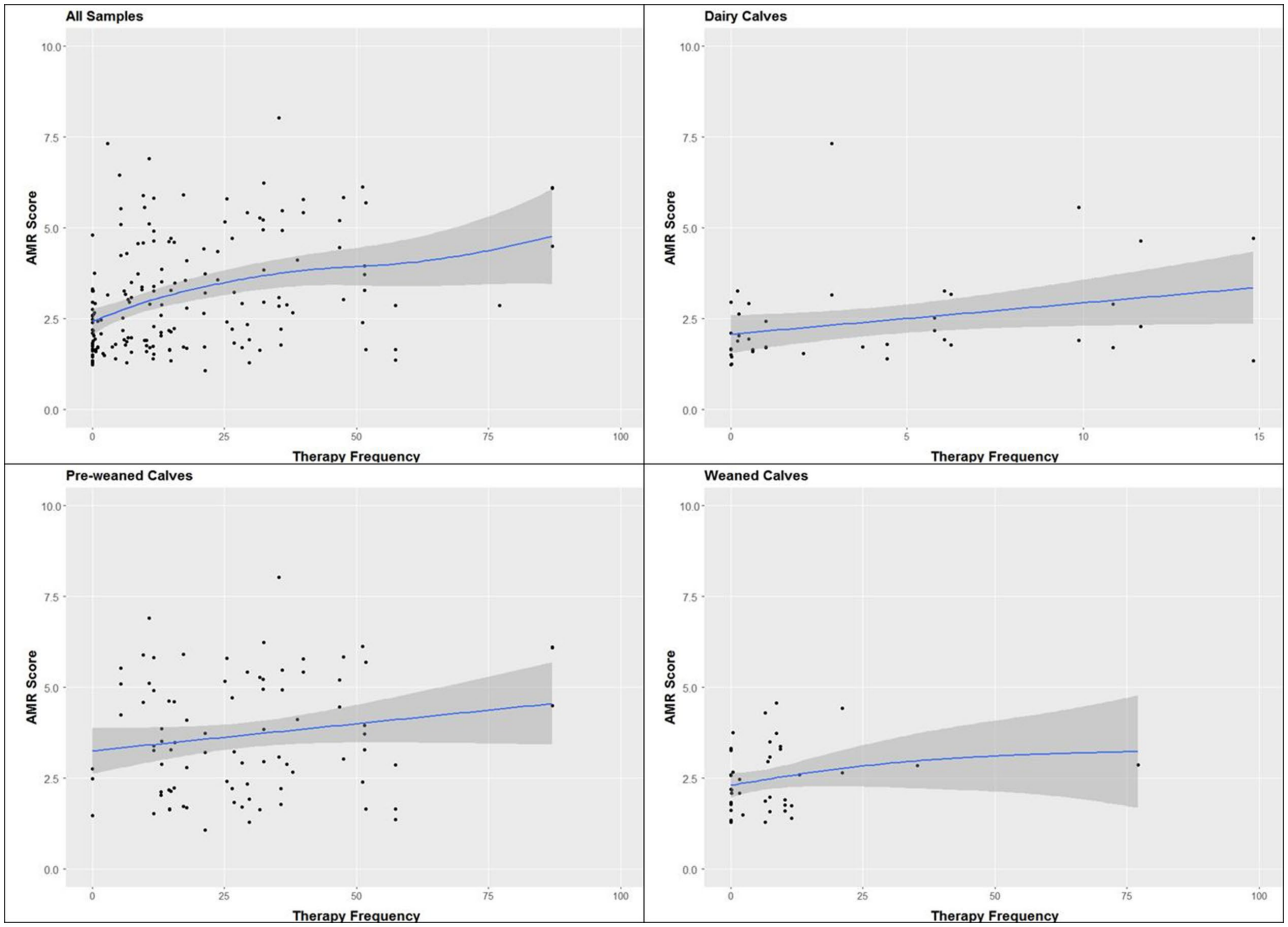
Association between the AMR score and the current therapy frequency: all farms, dairy farms, pre-weaned calf farms, weaned calf farms; *n* = 178 samples. Displayed is the estimate (blue line) and the 95% confidence intervals (dark gray area).

TF ranged from 0 to 87 (median 11.6) and was lowest in dairy farms (median 1.0) and highest in pre-weaned calf farms (26.5, weaned calf farms 6.5). The association between TF and AMR score was positive and more or less linear in all farm types as well as in the totality of data (Figure 5).

#### 3.3.5. Antimicrobial resistance score and age at housing in weeks

**Figure 6:**
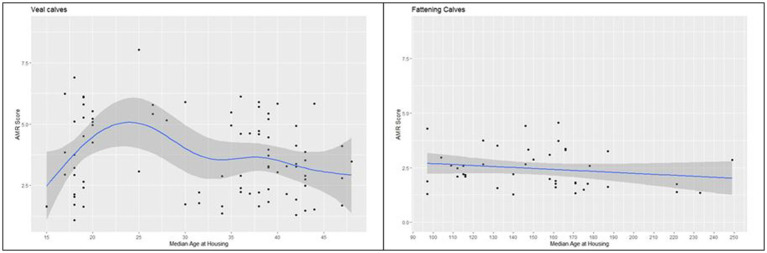
Association between AMR score and median age at purchase in days in farms with pre-weaned calves and weaned calves (*n* = 134 samples). Displayed is the estimate (blue line) and the 95% confidence intervals (dark gray area).

The association between age at purchase and AMR score was investigated separately for pre-weaned and weaned calf farms (Figure 6). Between 15 and 25 days of life, the AMR score increased but then slightly decreased.

## 4. Discussion

Our investigation showed that the use of antibiotics and the occurrence as well as rates of phenotypical resistance of *E. coli* in calf rearing sites were related. This finding correlates with the results of many other studies, not specifically in calf rearing farms, but in general animal husbandry as well as in human medicine (1, 3).

### 4.1. Levels of resistance

The high resistance rate against SMO is due to its use as a coccidiostat rather than its antimicrobial effect (25). This is in accordance with the EU Summary Report 2021 (3). SMO, TRP, and TET, the substances with the highest resistance rates, are used against enteritis and respiratory infections and can be applied via feed, which makes them a suitable group treatment. GEN is less commonly used because it can only be applied parenterally. AZM, CAZ, CIP, CTX, NAL, and TGC (only humans) are registered mainly for respiratory diseases, while those that can be administered orally are used for group treatments (AZM, CIP, and TGC). COL and AMP have only been registered for the treatment of enteric infections. The resistance rate to AMP was quite high, but that to COL was rather low. CMP has been banned for use in food-producing animals since 1994 (26). It is still routinely tested because the fluorinated derivative florfenicol is widely used against infections of the respiratory tract and can be applied as a long-acting preparation (27). The assessment of the substances used will be discussed elsewhere (28).

All resistance rates were slightly higher in our study compared to the German zoonosis monitoring in 2019 (14), with larger differences concerning GEN (9.7% vs. 2.8%) and CMP (24.3% vs. 10.1%), CTX (3.2 vs. 2.3%), CAZ (3.2 vs. 1.8%), NAL (10.3 vs. 5.5%), CIP (15.5 vs. 12.9%), AMP (39.7 vs. 33.6%), COL (2.1 vs. 0.5%), SMO (82.0% vs. 27.6%), TRP (34.3 vs. 27.2%), TET (49.0 vs. 37.3%), and AZM (5.4 vs. 2.8%). The differences might be due to the sampling material, because for zoonosis monitoring, cecal content from 217 slaughtered animals was investigated, while our study included 1,781 isolates from 178 pooled fecal samples. The investigation of 10 isolates per sample might have detected more levels of resistance than the single isolate per sample that was taken for zoonosis monitoring. The main objective of the zoonosis monitoring is to observe trends over time. Thus, the sample size per animal species and material is not high enough for a proper prevalence estimation.

Compared to the most recent studies on the national zoonosis monitoring, the percentage of completely susceptible isolates was lower in our study than in the recent zoonosis monitoring 13 a (pre-weaned calves), 18 (weaned calves), and 17% (dairy calves) vs. 42 (pre-weaned calves), 46 (weaned calves), and 78% (dairy calves) (29). On the other hand, 23% (95% confidence interval (CI) 13–29%; dairy calves), 42% (95%CI 33–51%, pre-weaned calves), and 36% (95% CI 32–41%, weaned calves) of the isolates in the resistance monitoring showed resistance to three or more substances, but 36% (95% CI 32–41%, dairy calves), 48% (95% CI 45–51%, pre-weaned calves), and 43% (95% CI 38–47%, weaned calves) of the isolates in our study. The 95% CI indicates substantial differences only for dairy farms, where the structures differ widely between farms, and many possible influencing factors, such as detailed husbandry conditions and the presence of other animals, were not investigated in the zoonosis monitoring.

### 4.2. Antimicrobial resistance score

According to previous studies (30–35), the highest AMR scores were observed in pre-weaned calf farms. This is not surprising because this animal group is at the highest risk of infectious diseases in general (33). Calves at the age of 14 days are transported and housed together in groups with calves from other farms, resulting in high infectious pressure. The calf’s immune system is not yet competent, but maternal antibodies from the colostrum are decreasing. Moreover, calves come from many different farms and are exposed to unknown pathogens against which neither maternal nor own antibodies are efficient. This, in combination with transport stress, increases the risk of infection. Calves that are already on the farm for a few weeks become immunocompetent, have adapted to the stable bacterial flora, and have less risk of being infected (36). Thus, the difference between the youngest and oldest animals was most significant in the pre-weaned calf farms, less in the dairy farms, and negligible in the weaned calf farms.

One weakness of the study was that the number of participating farms was different between the farm types; thus, within-group analyzes were not possible. It must also be considered that the actual age of the “youngest” and the “oldest” group differed between the farm types and even within the farm types so that general comparisons cannot be drawn. The effect of age should be assessed by the age at purchase, whereas the effect of the groups must be regarded in terms of infection pressure, as described above.

The analysis of the results from the hospital boxes was biased because the number of animals from which the collective fecal samples were derived varied and sometimes only included one animal. Because the diseases of the animals in the hospital box were not recorded, the heterogeneity of the health status in these hospital boxes might be large and cannot be assessed. This might explain the lack of elevated levels of resistance among the animals in the hospital boxes, as shown in other studies when diseased animals were treated with antibiotic substances (37, 38).

### 4.3. Therapy frequency and AMR score

The almost linear relationship between TF and resistance score proves the high association between both parameters and reflects that every use of antibiotics promotes the selection of resistant bacterial strains (30–33, 39–43). Any approach to reduce the resistance pressure in calf-keeping farms must address the need for antibiotic therapy. Thus, it is of utmost importance to improve animal health by reducing the risk of infectious diseases, especially in the period of the immunologic gap between maternal antibodies and their own immunocompetence. In Germany, a new regulation of animal transport came into force in 2022, which allows the transport of calves only from 28 days of life (44). The association between antibiotic use and median age at purchase is well known (31, 36) and is also explained by the high infection pressure and low immunocompetence of younger calves.

## 5. Conclusion

In conclusion, the regular use of antimicrobials in calves is associated with the release of AMR into the environment and to humans, and thus, is related to health. Further efforts are necessary to educate and motivate calf keepers to ensure the highest levels of hygiene and management, as well as animal welfare conditions, and to improve animal health (45). Some promising approaches are already published (32, 44–47).

## Data availability statement

The raw data supporting the conclusions of this article will be made available by the authors, without undue reservation.

## Author contributions

RM: conceived and designed the analysis, performed the analysis, and wrote the manuscript. SW: conceived and designed the analysis, collected the data, and performed parts of the analysis. LG: conceived and designed the analysis and collected the data. JB: contributed to the analysis. CR: conceived and designed the analysis and contributed to the analysis AF: conceived and designed the analysis and contributed to the analysis. KJ: performed parts of the analysis. All authors contributed to the article and approved the submitted version.

## Funding

This study was funded by the German Federal Ministry of Nutrition and Agriculture (grant number 2818HS014).

## Conflict of interest

The authors declare that the research was conducted in the absence of any commercial or financial relationships that could be construed as potential conflicts of interest.

## Publisher’s note

All claims expressed in this article are solely those of the authors and do not necessarily represent those of their affiliated organizations, or those of the publisher, the editors and the reviewers. Any product that may be evaluated in this article, or claim that may be made by its manufacturer, is not guaranteed or endorsed by the publisher.
